# Effect of plasma thrombin-antithrombin complex on ischemic stroke: a systematic review and meta-analysis

**DOI:** 10.1186/s13643-023-02174-9

**Published:** 2023-02-14

**Authors:** Peipei Song, Jianqin Xie, Wei Li, Xinying Zhang, Zhipeng Sun, Chongge You

**Affiliations:** 1 Laboratory Medicine Center, Lanzhou University Second Hospital, Lanzhou, 730000 China; 2 Department of Anesthesiology, Lanzhou University Second Hospital, Lanzhou, 730000 China; 3 Academic Department, Sysmex Shanghai Ltd., Shanghai, 200120 China

**Keywords:** Ischemic stroke, Prognosis, Stroke severity, Thrombin–antithrombin complex

## Abstract

**Background and objective:**

Thrombin-antithrombin complex (TAT) is a prethrombotic marker, and its application in ischemic stroke is still uncertain. The purpose of this systematic review and meta-analysis is to evaluate the relationship between plasma TAT and ischemic stroke base on the current evidence.

**Methods:**

A systematic literature search was conducted for searching the relative studies that investigated the association of TAT and ischemic stroke in PubMed, EMBASE, and Cochrane library databases. Mean difference and 95% confidence interval as the effect sizes were synthesized by random effects model in Review Manager (RevMan) Version 5.4. The heterogeneity was investigated using the chi-square test and the possible sources of heterogeneity were explored by sensitivity analysis and meta-regression. The publication bias was estimated by Egger’s tests.

**Results:**

A total of 12 eligible studies were included involving 1431 stroke cases and 532 healthy controls, of which six studies were eventually included in the meta-analysis. Plasma TAT in patients with ischemic stroke was significantly higher than that in healthy controls (MD 5.31, 95% CI = 4.12–6.51, *P* < 0.0001, *I*^2^ = 97.8%). There is a difference of TAT level in the same period among cardioembolic, lacunar, and atherothrombotic stroke (all *P* < 0.0001), in which the cardioembolic stroke with the highest level. Meanwhile, it is significant of TAT levels among various phases of cardioembolic stroke and the acute phase are markedly elevated (MD 7.75, 95CI%, 6.07–9.43, *P* < 0.001). However, no difference was found in the atherothrombotic (*P* = 0.13) and lacunar stroke (*P* = 0.34). Besides, the higher TAT level is closely related to the poor prognosis of patients with ischemic stroke, including higher recurrence, mortality, unfavorable recovery (modified Rankin scale > 2), and poor revascularization.

**Conclusions:**

This study suggested that plasma TAT levels are different in ischemic stroke subtypes, which are closely associated with the progression and might have an effect on the prognosis.

**Systematic review registration:**

PROSPERO CRD: 42021248787

**Supplementary Information:**

The online version contains supplementary material available at 10.1186/s13643-023-02174-9.

## Introduction

Stroke has become a serious threat to human health since it is one of the causes that lead to permanent disability and death worldwide [1]. More than 795,000 patients suffer from stroke and about 140,000 died every year in the USA (https://www.cdc.gov/stroke/facts.htm) [2]. Ischemic stroke accounts for 87% of total strokes and is the main focus of current stroke research [3]. Thrombosis is an important initiative of ischemic stroke, and serum D-dimer concentrations are commonly used to monitor thrombotic status, but specificity is still lacking. Therefore, prospective studies of earlier biomarkers measured at different stages of ischemic stroke are more likely to help identify therapeutic and prognostic mechanisms.

Ischemic brain damage involves the activation of a series of detrimental signaling cascades, including micro-atheroma, endothelial dysfunction, and inflammation. There is no simple and effective method for early detection other than the detection of narrowed blood vessels by imaging techniques. So, the clinical guidelines suggest that thrombolysis is the most important method in the emergency department under the instruction of multi-mode computed tomography (CT) or magnetic resonance imaging (MRI) neuroimaging once an ischemic stroke occurs [4]. However, the treatment of intravenous thrombolysis with recombinant tissue thrombolytic plasminogen activator (rt-PA) window-time is very narrow, usually within 3.0–4.5 h after the onset of neurologic symptoms, which delays the diagnostic time for visible neuroimaging techniques [5, 6]. On the other hand, it is difficult to distinguish ischemic stroke subtypes from these similar focal neurological deficits, such as complex migraine, demyelinating diseases, and vascular diseases [7]. In addition, the utility of advanced MRI technology current is limited because of lacking available emergency services or vascular neurologists [8]. Therefore, it is essential to find some simple biomarkers that can distinguish ischemic stroke subtypes and stages.

Research on some easily available blood biomarkers in ischemic stroke has been reported in a previous review for the diagnosis and differentiation of stroke, including brain natriuretic peptide, matrix metalloproteinase-9, and D-dimer [9]. Thromboembolism and situ thrombosis are the main causes of blood circulation blockage in ischemic stroke, which all involve disorders of the coagulation system. D-dimer, a cross-linked fibrin clot degradation product, is one of the biomarkers of the fibrinolysis process. In contrast, thrombin binds to antithrombin III (AT-III) to form the thrombin-antithrombin (TAT) complex earlier, and TAT is also more easily detected than readily degradable thrombin. Therefore, TAT is considered to be a biomarker for the early stages of coagulation activation, which means that thrombin is formed and enters a prothrombotic state. However, the effect of TAT on ischemic stroke is not fully clear.

Elevated TAT has been proven to be associated with several disease studies. There is clear evidence that patients with myocardial infarction were accompanied by mild inflammation with an increase in TAT [10]. The activity of TAT could identify patients with ongoing severe coagulopathy early in the course of sepsis [11]. Determination of the plasma TAT complex concentration is helpful for the evaluation of the prognostic severity of post-ICH brain injury [12]. Since ischemic stroke is a dynamic process, in which different degrees of activation of coagulation exist in the various phases associated with distinct biological substrates molecular [13]. However, to our knowledge, there are few reports on the comprehensive analysis of TAT in patients with ischemic stroke, and the association between TAT and the stage or subtypes of the disease is unclear. Hence, defining the ideal blood biomarker is of clinical importance for precision medicine in deciding stroke-type specific treatment.

Therefore, we performed a meta-analysis to clarify whether there are differences in TAT between ischemic stroke subtypes and different responses by splitting the analysis on the timing of the blood draw with ischemic stroke by reviewing the literature. Besides, we did a systematic review to explain the relationship between TAT levels and prognosis reported in the patient groups for helping reduce mortality and better beyond hospital admission management.

## Methods

We performed this systematic review and meta-analysis according to the Preferred Reporting Items for Systematic Reviews and Meta-Analyses (PRISMA) [14]. The protocol of this study has been registered in the International Prospective Register of Systematic Reviews (PROSPERO CRD: 42021248787).

### Literature search

A comprehensive search was conducted by two researchers (Peipei Song and Jianqin Xie) from inception to October 13, 2021, in PubMed, EMBASE, Cochrane Library, Web of Science, and Google Scholar. The combination of MeSH and free text terms were used to search in the databases and the strategy of PubMed as follow: (“antithrombins” OR “thrombate III” OR “AT III-protease complex”) AND (“stroke” OR “cerebral infarction” OR “cerebrovascular accident”). The search results were limited to human studies that were written in English, and we also manually searched the references of original articles and reviews. Two authors independently completed the process of literature search and any indistinguishable records were conducted by negotiating with the third party (Chongge You). We showed the database detailed search strategies for the databases in Supplementary Table 1.

### Inclusion and exclusion criteria

All studies met the following inclusion criteria and were selected into our systematic review and meta-analysis based: (1) case-control study or cohort study, (2) study investigated the association of plasma TAT levels and strokes, (3) study including stroke subtypes or stages, (4) plasma TAT as one of the outcome indicators was reported in prognosis, (5) if the studies with the same case materials and published by the same authors, only the lasted or largest population will be included. (6) We do not limit the period and language of included studies.

The studies were excluded in which they meet the following exclusion criteria: (1) repeated publications, (2) reviews or summaries, (3) non-human studies, (4) incomplete data in the research, and (5) studies measuring TAT level in cerebrospinal fluid or urine.

### Definition of subgroups

Cardioembolic, lacunar, and atherothrombotic stroke subjects were divided based on the findings of the clinical ancillary examination, such as cerebral angiography, ultrasound examination of carotid vessels, and confirmation of the intracardiac embolization and the evidence of image logical diagnosis. We defined the acute phase (< 24 h), subacute phase (24–48 h), and chronic phase (> 7 days) as the records. Another subgroup was determined according to the methods of TAT measurement.

### Data extracted and assessment of the quality

Two authors (Wei Li and Xinying Zhang) independently completed the data extraction and quality assessment. For any indistinguishable record by consulting or talking over the corresponding author. We read the full text for extracting data and further assessment. The baseline characteristics of the literature were extracted by using a well-designed form. The Newcastle-Ottawa Scale (NOS) scale was used to assess the methodological quality of those included the case-control or cohort studies [15]. Each of the literature is scored based on eight questions in three main broad categories: (1) patient selection; (2) comparability of study groups; (3) assessment of exposure. The total score (TS) is 9, and the TS ≤ 5, 6 ≤TS ≤ 7, and TS ≥ 8 were regarded as low, moderate, and high quality respectively.

### Statistical analysis

Two authors (Peipei Song and Jianqin Xie) used the software of StataMP version 16 to synthesize study data, and *P* < 0.05 is considered to be statistically significant. We calculated the means and standard deviations in line with the method developed by Hozo et al. [16] if the article provided the medians and ranges instead of the means and standard deviations. Mean difference (MD) was calculated as a summary statistic in the random-effects model (Mantel–Haenszel test). The statistical heterogeneity was considered when *P* ≤ 0.05 in comparison among groups or *P* < 0.1 in comparison within groups. Chi-square test was used to assess the heterogeneity among the included studies, and quantified by the Higgin’s *I*^2^ statistic, *I*^2^ < 50%, 50% ≤ *I*^2^ ≤ 75%, and *I*^2^ > 75% were regarded as low, moderate, and high heterogeneity, respectively. Subsequently, a sensitivity analysis was performed when there is the heterogeneity, in which one study at a time was removed and the others analyzed to estimate whether the results could have been affected markedly by a single study. Furthermore, an analysis was performed by meta-regression to searched for sources of heterogeneity. Subgroup analysis was performed according to the different subtypes and phases of ischemic stroke. Egger’s regression tests were used to estimate the publication bias.

## Results

### Characteristics of included studies

**Fig. 1:**
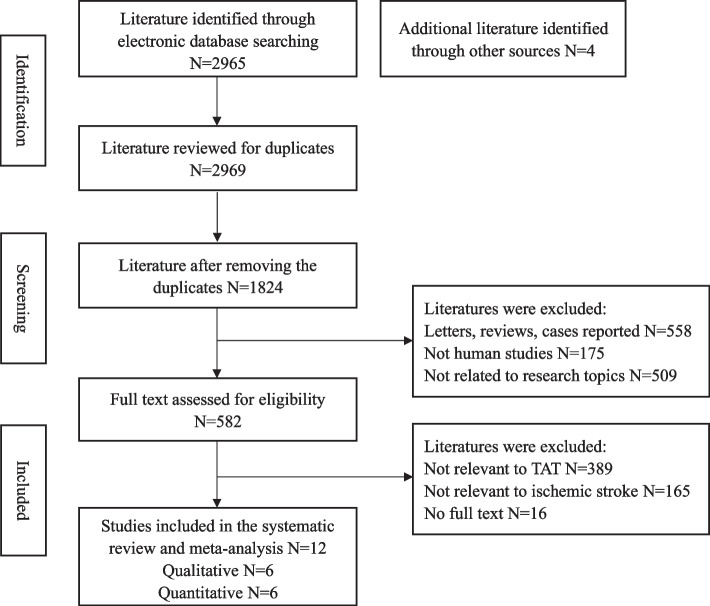
PRISMA flow diagram for the detailed procedures of study screening of systematic review and meta-analysis

After retrieving relevant search terms in PubMed, EMBASE, Web of Science, and Cochrane Library databases, a total of 2965 publications were researched, and 4 articles were retrieved from Google Scholar. The PRISMA flow diagram for study inclusion in our meta-analysis was depicted in Fig. 1. 1824 articles remaining after deduplication, and then the letters, reviews, cases reported, animal studies, and literature not related to the topic totaling 1242 publications were excluded by browsing the titles and abstracts. A full text of 582 potentially eligible articles was retrieved, studies were excluded again for no relation with TAT (389), study topic is not a stroke (165), and without full text (16). Finally, 12 studies were included in our meta-analysis.

A total of ten case-control studies and two cohort studies were incorporated into the systematic review and meta-analysis, including 1431 stroke cases and 532 healthy controls. Six studies were from Japan [17–22], two studies were from China [8, 23], and the remaining four were from Canada [24], the USA [25], Spain [26], and Finland [27]. The average ranges from 64.7 to 72.4 years old in the case group and 61 to 72.4 in the control. Computed tomography (CT) and/or magnetic resonance imaging (MRI) are the methods used in all studies to confirm the diagnosis. Serum TAT levels were measured by enzyme-linked immunosorbent assay kits (ELISA) in nine studies, two studies used enzyme immunoassays to determine the biomarker, and only one study was detected by chemiluminescence. Six of the studies conducted follow-up records (1 month to 3 years) and reported the cut-off of TAT, three of which explained the OR between TAT and adverse outcome. Table 1 summarizes the characteristics of included studies, and the follow-up results are shown in Table 2.

**Table 1:** Characteristics of included studies in the systematic review meta-analysis

Studies	Patients	Country	Confirmed method	Cases	Average age	Control	Average age	Test time	Test method	From first onset to hospital
Kentaro Takano 1991 [17]	Acute cardioembolic stroke	Japan	2D-echo, CT	22	64.70	25	61.00	< 24 h	ELISA	Within 24 h
Ran Meng 2011 [8]	Cerebral ischemic stroke	China	MRI/DWI/MRA or CTA	152	58.72	46	51.89	< 24 h	ELISA	Within 4.5 h
Fon E A 1994 [24]	Transient ischemic attack (TIA)	Canada	CT	36	68.80	65	65.50	< 7 days, 1 months, 3 months	ELISA	Within 7 days
Ye Naifang 2020 [23]	Acute ischemic stroke	China	CT and/or MRI	236	70.00	90	69.00	< 12 h	Chemilumi-nescence	Within 24 h
Israel Fernandez Cadenas 2009 [26]	Ischemic stroke	Spanish	CT and MRI	89	72.40	–	–	Admission, 1 h, 2 h, 24 h, 48 h after post-tPA	ELISA	Within 3 h
Haapaniemi E 2004 [27]	Ischemic stroke	Finland	radiologically	55	60.20	55	60.00	2 days, 7 days, 1 months, 3 months	ELISA	Within 24 h
Satoshi Kataoka 2000 [18]	Acute brain infarction	Japan	CT and MRI	137	68.32	32	66.50	48 h, 1 week, 3 weeks	ELISA	Within 48 h
Yamazaki M 1993 [19]	Cerebral infarction	Japan	CT or MRI	138	65.40	23	60.00	7 days, 1 months, and in 1 months	EIA	No description
Noriko Ono 1991 [20]	Ischemic stroke	Japan	CT and MRI	98	69.50	50	66.00	< 24 h	ELISA	Within 5 days
Masao Nagayama 1994 [21]	Ischemic stroke	Japan	CT	53	63.50	37	60.00	48 h, 3 days 7 days, 14 days, 1 months	ELISA	Within 3 days
Takano K 1992 [22]	Acute ischemic stroke	Japan	CT and cerebral angiography	54	66.00	20	61.60	48 h, 7 days, 1 mon	ELISA	Within 48 h
David Tanne 2006 [25]	Acute ischemic stroke	USA	CT	361	68	–	–	2 h, 24 h, 7–10 days, and 3 months	EIA	Within 24 h

*CT* computed tomography, *MRI* magnetic resonance imaging, *2D-echo* two-dimensional echocardiography, *DWI* diffusion-weighted imaging, *PWI* perfusion-weighted imaging, *ELISA* enzyme-linked immunosorbent assay, *EIA* enzyme immunoassays

**Table 2:** The relationship between TAT and the outcome of ischemic stroke patients after a follow-up

Studies	Patients	Country	Cases	Average age	From the first onset to hospital	Cut-off of TAT	Follow-up time	Recurrence cases	Outcomes	OR	95CI	Test method
Takano K 1991 [17]	Acute cardioembolic stroke	Japan	22	64.7	Within 24 h	12.1 ng/mL	2 months	8	Recurrence	–	–	ELISA
E A Fon 1994 [24]	Transient ischemic attack (TIA)	Canada	36	68.8	Within 7 day	7.8 ng/mL	13 months	12	Recurrence	–	–	ELISA
Haapaniemi E. 2004 [27]	Ischemic stroke	Finland	55	60.2	Within 24 h	7.9 ng/L	3 years	16	Recurrence	–	–	ELISA
David Tanne 2006 [25]	Acute ischemic stroke	USA	361	68.0	Within 24 h	11.2 ng/mL	3 months	–	Mortality	1.72	1.26–2.34	EIA
Ye Naifang 2020 [23]	Acute ischemic stroke	China	236	70.0	Within 24 h	6.03 ng/mL	1 months	35	Unfavorable function	1.283	1.105–1.489	Chemilumi-nescence
Israel Fernandez-Cadenas 2009 [26]	Ischemic stroke	Spanish	89	72.4	Within 3 h	< 24 μg/L	3 months	16	Revascularis-ation rates	2.7	1.2–6.1	ELISA

*ELISA* enzyme-linked immunosorbent assay, *EIA* enzyme immunoassays

### Quality of included studies

All studies were assessed for methodological quality by the Newcastle-Ottawa Scale (NOS). The range of quality scores is 7 to 8 for case-control, with a median of 7.5 (7.5 ± 0.53) (Supplementary Table 2). All studies reported the case and control definition, comparability, ascertainment of exposure, and the same method of ascertainment for cases. But five of them do not report the source of the control group [17–19, 21, 22]. All the research included a certain hospital or institution for a continuous-time, which we believe to be representativeness of the cases. However, no study describes the non-response rate. The quality scores of the two cohort studies were 6 [26] and 7 [25] respectively, both neither described comparability and non-exposed cohort, and one of which has no statement about adequacy of follow-up of the cohort [26].

### Meta-analysis

**Fig. 2:**
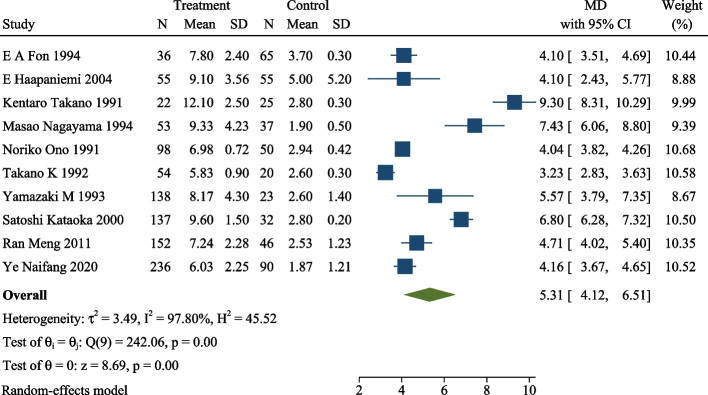
Plasma TAT levels in ischemic stroke and healthy control

Ten studies investigated the association between plasma TAT levels and ischemic stroke. The result revealed that plasma TAT level was significantly higher in ischemic patients than those in healthy controls (MD 5.31, 95% CI = 4.12–6.51, *P* < 0.0001, *I*^2^ = 97.8 %, *P*_heterogeneity_ < 0.00001, Fig. 2).

**Fig. 3:**
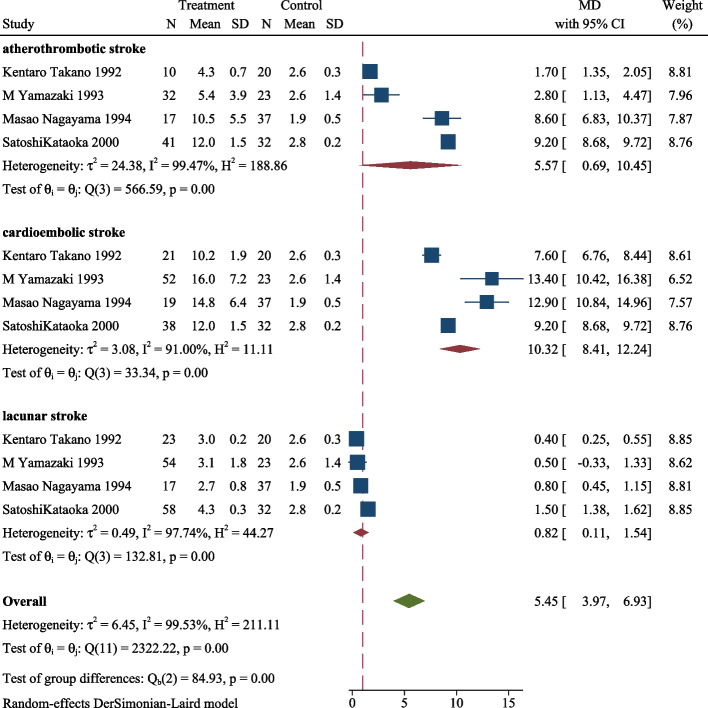
Plasma TAT levels in the acute phase of cardioembolic, lacunar, and atherothrombotic strokes

**Fig. 4:**
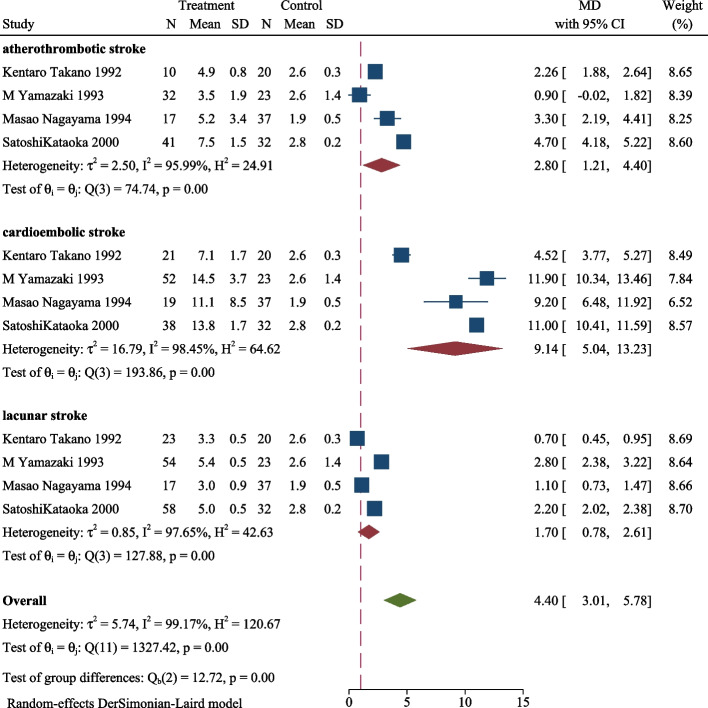
Plasma TAT levels in the subacute phase of cardioembolic, lacunar, and atherothrombotic strokes

**Fig. 5:**
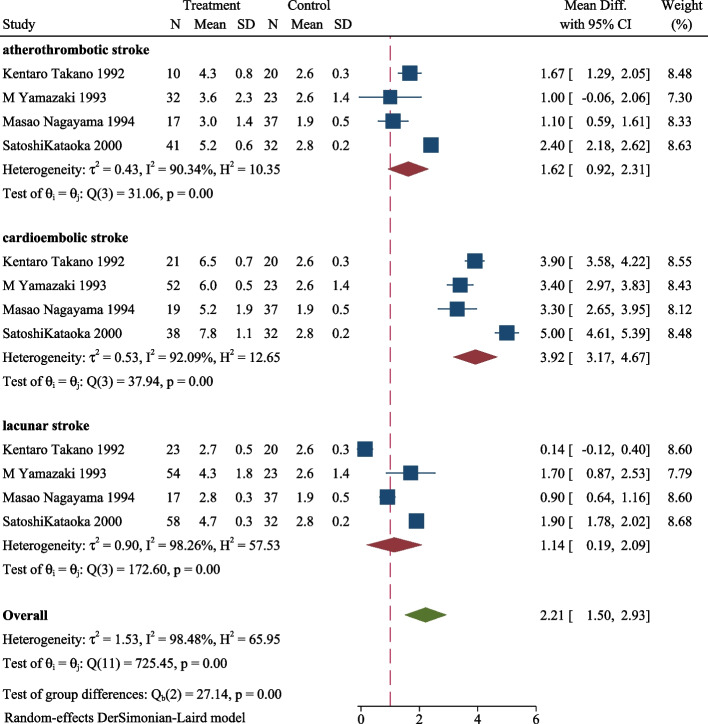
Plasma TAT levels in the chronic phase of cardioembolic, lacunar, and atherothrombotic stroke

We conducted a subgroup analysis to explore the distinction in the same period among cardioembolic, lacunar, and atherothrombotic strokes and the results are shown in Figs. 3, 4, 5. The plasma TAT level in the three subgroups of ischemic stroke was significantly higher than control in the acute phase (MD 5.45, 95CI%, 3.97–6.93, *P* < 0.001, Fig. 3). Similarly, the ischemic stroke subgroup with a higher level of TAT than the control in subacute phases (MD 4.40, 95CI%, 3.01–5.78, *P* < 0.001, Fig. 4), and chronic phase (MD 2.21, 95CI%, 1.50–2.93, *P* < 0.001, Fig. 5). During the same period, the plasma TAT level of cardioembolic stroke is the highest, followed by atherosclerosis and lacunar stroke.

Meanwhile, we analyzed the differences among different periods in each subtype of ischemic stroke. The cardioembolic stroke was shown in Supplementary Figure 1, it is significant in the acute, subacute, and chronic phases (MD 7.75, 95CI%, 6.07–9.43, *P* < 0.001). Although there is no difference in atherothrombotic stroke in the result of Supplementary Figure S2 (MD 3.26, 95CI%, 2.02–4.50, *P* = 0.13), and the plasma TAT level was markedly elevated in the acute than that in the subacute and chronic phases. Besides, no significant difference was found in the lacunar stroke which was shown in Supplementary Figure S3 (MD 1.22, 95CI%, 0.79–1.65, *P* = 0.34*, I*^2^ = 97.86%), and the highest plasma TAT level is the subacute phase.

We further searched for sources of heterogeneity by meta-regression, and these results are showed in the Supplementary Table 3. The moderators included year, country, cases, average age, and test method, unfortunately, none of them were sources of heterogeneity.

### Systematic review

A total of six studies have reported the relationship between plasma TAT levels and the prognosis of ischemic stroke prognosis (Table 2). We conducted the systematic review because the available data did not further perform synthesis. Three studies recorded recurrence of stroke, the incidence of short-term recurrence was 33%, which was associated with a mean TAT concentration of 9.6 ng/mL [17, 24, 27]. David Tanne et al. [25] found that the baseline TAT level is related to three-month mortality when it is more than 11.2 ng/mL (OR = 1.72, 95% CI 1.26–2.34). Similarly, the study of Israel Fernandez et al. [23] indicates that there was a significant correlation between higher TAT levels and unfavorable function outcomes (poor outcome modified Rankin scale > 2) (OR = 1.283, 95% CI 1.105–1.489). On the contrary, lower plasma TAT levels in patients with ischemic stroke could be associated with a high success rate of revascularization (OR = 2.7, 95% CI 1.2–6.1) [26].

### Sensitivity analysis and publication bias

Sensitivity analysis was performed through the elimination method in which one study is removed at a time and the others analyzed to estimate whether the results could have been affected markedly by a single study. However, the results showed that excluding any one study will not affect the results, this analysis confirmed the stability of the results. Another subgroup analysis was performed to explore the source of heterogeneity based on the TAT detection method. There was no significant difference between the groups (*P* = 0.11, Supplementary Figure S4). There is no publication bias which was estimated by Egger’s (*P* = 0.7787) regression test.

## Discussion

This systematic review and meta-analysis analyzed the relationship between plasma TAT and ischemic stroke. The results showed that there is a higher level of TAT in the ischemic stroke group than healthy control. Meantime, the plasma TAT level will liner decrease with the progress of ischemic time, and with the highest in the acute phase. Compare with atherothrombotic and lacunar stroke, a higher concentration of TAT was found in cardioembolic stroke. The result of this systematic review showed that plasma TAT was closely related to the subsequent prognosis.

The increasing occurrence of ischemic stroke was reported in recent years as the population ages [28]. Although recent clinical trials have shown that there are many opportunities to improve stroke prevention strategies and effectively intervene in acute strokes, there are still challenges in early and rapid diagnosis. CT or MRI are commonly used in clinical diagnosis at current, but the confirmation time of lesions far exceeds the optimal treatment period. Patients admitted within four hours were applied to thrombolytic therapy recommended by clinical guidelines, which is an effective treatment [6]. Besides, no specific markers have been found that can be used for the early diagnosis of ischemic stroke. The essence of ischemic stroke is a thrombotic disease caused by endothelial injury, hypercoagulation, or blood flow disorder. Thrombus can be classified into thromboembolism and thrombosis according to the different sources of embolus. Therefore, ischemic stroke main included atherosclerotic and cardioembolic strokes, which are caused by thrombosis and thromboembolism, respectively. Another lacunar stroke is also called asymptomatic cerebral infarction, usually a small blood vessel obstruction of no more than 1.5 cm in diameter [29]. TAT is a complex of thrombin and antithrombin, most circulating TAT derives from the process of thrombosis caused by endothelial cell injury. TAT can be detected in thrombus-related diseases since it indicates the activation of thrombin which contributes to thrombus generation [30]. Hence, it is meaningful to find biomarkers with diagnostic value.

In our study, the results demonstrated that patients with cardioembolic stroke have higher TAT levels than those with atherothrombotic strokes, which suggested that TAT might be able to differentiate cardioembolic stroke from atherothrombotic (*P* = 0.006). Similar to our result, Song, J et al. proved that cardioembolic stroke might be severer and with higher TAT levels than other stroke subtypes [31]. The TAT in subtypes of ischemic stroke differs depending on the underlying mechanism of embolus formation. Thrombosis in the coronary artery or heart cavity caused by rupture or fissuring of plaques or damage to the heart structure will flow and block the cerebral vessels, especially in atrial fibrillation [32]. Besides, compared with cerebrovascular, plentiful large clotting factors were activated in the heart blood vessel, more blood cells are recruited and a large amount of fibrinogen is activated, and then thrombi are formed. Some studies have shown that the difference between stroke thrombus and acute myocardial infarction thrombus lies in the abundance of platelets and fibrin and the increase in the number of inflammatory cells per thrombus area [33, 34]. However, in atherosclerotic stroke, the plaques that are most prone to rupture with a rich lipid core and thin fibrous cap, inflammatory cells accumulate at the site of endothelial destruction are the main driving factor for the growth of early pale thrombus. There have been a few studies reporting patients with carotid atherosclerosis did have a better functional outcome than patients with cardioembolism [35].

Previous studies have investigated the association between TAT and acute myocardial infarction (AMI), which showed that the highest TAT level is at the subacute phase for those patients with myocardial infarction [10, 36]. However, a higher TAT level in the acute phase of ischemic stroke was found in our result (MD 5.45, 95CI%, 3.97–6.93, *P* < 0.001), which might be related to the mechanism of thrombus formation in the different diseases. This is most commonly due to thrombus formation in a coronary artery [34]. The present findings demonstrated different sequential alterations in thrombotic markers following three subtypes of brain infarct [37]. Thrombosis in large blood vessels is not easy to detect in the early stage, and there are usually some confusing signs before the occurrence of acute myocardial infarction, such as arrhythmia and angina pectoris. TAT levels in the subacute phase may also be affected by mechanical treatment, with more thrombin production induced by the stimulation of endothelial injury. However, no matter thrombosis or thromboembolism, the cerebral blood flow will be blocked instantly in patients with cerebral infarction, leading to hypoperfusion and clinical symptoms. In addition, asymptomatic focal small cerebral vascular embolism is also common in ischemic strokes, such as lacunar infarction.

Several published systematic reviews have generated a shred of evidence regarding the role of biomarkers in ischemic stroke. Yan H et al. investigated the correlations of TM and hs-CRP with ischemic stroke [38]. A large number of articles have reported the D-dimer and its application in ischemic stroke [39], which is generally regarded as an indicator for evaluating thrombosis. In our study, the higher plasma TAT level was found in the subtype of the cardioembolic (MD 7.75, 95CI%, 6.07–9.43, *P* < 0.001) and acute phases (MD 5.45, 95CI%, 3.97–6.93, *P* < 0.001). For those patients admitted to the emergency department, higher plasma TAT levels may alert the clinician that he is more prone to cardiac infarction or earlier stages of disease. Besides, we found that higher TAT levels often indicate higher recurrence [27], worse functional recovery [23], and higher mortality [25]. This has important guiding value for the prognosis of recurrence and recovery of body function, and may be an independent influencing factor for monitoring thrombolytic status after therapy. Lower plasma TAT levels in ischemic stroke patients with thrombolytic tPA or mechanical recanalization therapies were more beneficial to successful vascular recanalization, which may be related to the activity of thrombin and the loose thrombus structure [26, 40]. Though plasma TAT showed the clinical value as an auxiliary factor to identify ischemic stroke subtypes and phases, it still needs to be verified in large-scale research.

If plasma TAT can be widely developed as one of the physical examination items, it is also a good measure to prevent thrombotic diseases. Currently, the study of comprehensive biomarkers in the stages of ischemic stroke is still in its infancy [41]. The changes in coagulation biomarkers may precede the imaging manifestations of clinical symptoms. Although our study showed the plasm TAT was beneficial for ischemic stroke, the specificity of TAT needs to be verified in other prethrombotic diseases. These biomarkers are unlikely to replace imaging tests as a first-line diagnostic tool for the detection of ischemic stroke, but they may be a strategy to reduce the cost of screening and as a mechanistic pathway aimed at new treatments. Use a combination of cheaper and high-sensitivity diagnostic tests, such as TAT and D-dimer, as the initial stage detection and followed by MRI tests. For developing countries especially where neuroimaging facilities are limited, or in settings where the brain imaging is normal, the use of blood biomarkers at the point of care in combination with the clinical predictors could be an effective alternative for rapid diagnosis of ischemic stroke in the future.

## Conclusion

Our meta-analysis results showed an important effect of plasma TAT levels on ischemic stroke, and plasma TAT might be involved in the progression and prognosis of ischemic stroke, and it is also helpful in identifying stroke subtypes.

## Limitation

We acknowledge limitations in our study. First of all, the number of studies included in our meta-analysis is small, and there are large differences in sample sizes, which may have an impact on statistical analysis and become another source of heterogeneity. Secondly, manual search for relevant reviews may lead to omissions in the literature, which may potentially affect the validity of our results. Finally, due to the lack of research data, the relationship between the severity of ischemic stroke based on the NIHSS scores and TAT has not been analyzed in this meta-analysis, which may be a part worth exploring in the future. Choosing English language articles only is also one of our shortcomings.

## Supplementary Information

**Additional file 1: Supplementary Table 1.** Search strategy for databases.

**Additional file 2: Supplementary Table 2.** The quality of included case-control studies.

**Additional file 3: Supplementary Table 3.** Meta-regression analysis of each study attribute.

**Additional file 4: Supplementary Figure S1**. The level of TAT in cardioembolic stroke among the acute, subacute and chronic phases.

**Additional file 5: Supplementary Figure S2.** The level of TAT in atherothrombotic stroke among the acute, subacute and chronic phases.

**Additional file 6: Supplementary Figure S3.** The level of TAT in lacunar stroke among the acute, subacute and chronic phases.

**Additional file 7: Supplementary Figure S4.** Subgroup analysis of plasma TAT based on the TAT detection method.

## Data Availability

There is no data applicable to the current protocol. The data extracted and analyzed in the prospective review will be made available from the corresponding author on request.
