# Supplementary material for: Effect of plasma thrombin-antithrombin complex on ischemic stroke: a systematic review and meta-analysis
Source: Syst Rev. 2023 Feb 14;12:17. doi: 10.1186/s13643-023-02174-9 (PMC9930276; doi:10.1186/s13643-023-02174-9)
Supplement: Supplementary file 1 — Additional file 1: Supplementary Table 1. Search strategy for databases. [file 13643_2023_2174_MOESM1_ESM.pdf]

Supplementary Table 1

Search strategy for Pubmed

Serial Number	Strategy
#1	"antithrombin III-protease complex"[Supplementary Concept] OR "Antithrombin III"[MeSH Terms]
#2	"Antithrombin"[Title/Abstract] OR "antithrombin III-protease complex"[Title/Abstract] OR "Heparin Co Factor I"[Title/Abstract] OR "Atenativ"[Title/Abstract] OR "Thrombate III" [Title/Abstract] OR "Antithrombin III Alpha"[Title/Abstract] OR "Kybernin"[Title/Abstract] OR "thrombin-antithrombin complex"[Title/Abstract] OR "AT III-protease complex"[Title/Abstract]
#3	#1 OR #2
#4	"Stroke"[Mesh]
#5	"Stroke"[Title/Abstract] OR "Cerebrovascular Accident"[Title/Abstract] OR "Cerebrovascular Apoplexy"[Title/Abstract] OR "Brain Vascular Accident"[Title/Abstract] OR "Cerebrovascular Stroke"[Title/Abstract] OR "Apoplexy"[Title/Abstract] OR "Cerebral Stroke"[Title/Abstract] OR "Acute Stroke"[Title/Abstract] OR "Acute Cerebrovascular Accident"[Title/Abstract] OR "Brain Infarction"[Title/Abstract] OR "Cerebral Infarction"[Title/Abstract] OR "Ischemic Stroke"[Title/Abstract] OR "Embolic Stroke"[Title/Abstract] OR "Thrombotic Stroke"[Title/Abstract] OR "Cardioembolic stroke"[Title/Abstract] OR "Lacunar stroke"[Title/Abstract] OR "Atherothrombotic stroke"[Title/Abstract] OR "Cryptogenic stroke"[Title/Abstract]
#6	#4 OR #5
#7	#3 AND #6

Search strategy for EMBASE

Serial Number	Strategy
#1	'antithrombin iii'/exp
#2	'antithrombin iii':ab,ti OR 'antithrombin protein':ab,ti OR 'heparin co factor i':ab,ti OR 'atenativ':ab,ti OR 'thrombate iii':ab,ti OR 'antithrombin iii alpha':ab,ti OR 'kybernin':ab,ti OR 'thrombin-antithrombin complex':ab,ti OR 'at iii-protease complex':ab,ti OR 'antithrombin iii-protease complex':ab,ti
#3	#1 OR #2
#4	'cerebrovascular accident'/exp
#5	'cerebrovascular accident':ab,ti OR 'stroke':ab,ti OR 'acute cerebrovascular lesion':ab,ti OR 'acute focal cerebral vasculopathy':ab,ti OR 'acute stroke':ab,ti OR 'apoplectic stroke':ab,ti OR 'apoplexi':ab,ti OR 'apoplexy':ab,ti OR 'brain blood flow disturbance':ab,ti OR 'brain accident':ab,ti OR 'brain attack':ab,ti OR 'brain insultus':ab,ti OR 'brain ischaemic attack':ab,ti OR 'brain ischemic attack':ab,ti OR 'brain vascular accident':ab,ti OR 'cerebral apoplexia':ab,ti OR 'cerebral insult':ab,ti OR 'cerebral stroke':ab,ti OR 'cerebral vascular accident':ab,ti OR 'cerebral vascular insufficiency':ab,ti OR 'cerebro vascular accident':ab,ti OR 'cerebrovascular arrest':ab,ti OR 'cerebrovascular failure':ab,ti OR 'cerebrovascular injury':ab,ti OR 'cerebrovascular insufficiency':ab,ti OR 'cerebrovascular insult':ab,ti OR 'cerebrum vascular accident':ab,ti OR 'cryptogenic stroke':ab,ti OR 'cva':ab,ti OR 'ischaemic cerebral attack':ab,ti OR 'ischaemic seizure':ab,ti OR 'thrombotic strokes':ab,ti OR 'brain infarction':ab,ti OR 'hemorrhagic stroke':ab,ti OR 'ischemic stroke':ab,ti OR 'cerebral infarction':ab,ti OR 'embolic stroke':ab,ti
#6	#4 OR #5
#7	#3 AND #6

Search strategy for Cochrane library

Serial Number	Strategy
#1	MeSH descriptor: [Antithrombins] explode all trees
#2	(antithrombin):ti,ab,kw OR ("Kybernin"):ti,ab,kw OR (Thrombate III):ti,ab,kw OR (Atenativ):ti,ab,kw OR (Heparin Co Factor I):ti,ab,kw
#3	(antithrombin III-protease complex):ti,ab,kw OR (thrombin-antithrombin complex):ti,ab,kw OR (AT III-protease complex):ti,ab,kw
#4	#1 OR #2 OR #3
#5	MeSH descriptor: [Stroke] explode all trees
#6	(Stroke):ti,ab,kw OR (cerebrovascular accident):ti,ab,kw OR (Cerebrovascular Apoplexy):ti,ab,kw OR (Brain Vascular Accident):ti,ab,kw OR (Cerebrovascular Stroke):ti,ab,kw
#7	(thrombotic stroke):ti,ab,kw OR (Apoplexy):ti,ab,kw OR (Cerebral Stroke):ti,ab,kw OR (Cerebral Stroke):ti,ab,kw OR (Acute Cerebrovascular Accident):ti,ab,kw
#8	(Brain Infarction):ti,ab,kw OR (hemorrhagic stroke):ti,ab,kw OR (ischemic stroke):ti,ab,kw OR (cerebral infarction):ti,ab,kw OR (Embolic Stroke):ti,ab,kw
#9	(Acute Stroke):ti,ab,kw OR (Cardioembolic stroke):ti,ab,kw OR (Lacunar stroke):ti,ab,kw OR (Atherothrombotic stroke):ti,ab,kw OR (Cryptogenic stroke):ti,ab,kw
#10	#5 OR #6 OR #7 OR #8 OR #9
#11	#4 AND #10
