# Supplementary material for: Effect of plasma thrombin-antithrombin complex on ischemic stroke: a systematic review and meta-analysis
Source: Syst Rev. 2023 Feb 14;12:17. doi: 10.1186/s13643-023-02174-9 (PMC9930276; doi:10.1186/s13643-023-02174-9)
Supplement: Supplementary file 2 — Additional file 2: Supplementary Table 2. The quality of included case-control studies. [file 13643_2023_2174_MOESM2_ESM.pdf]

Supplementary Table 2 The quality of included Case-Control Studies.

			Kentaro Takano 1991	Ran Meng 2011	E A Fon 1994	Ye Naifang 2020	E. Haapaniemi 2004	Satoshi Kataoka 2000	M Yamazaki 1993	Noriko Ono 1991	Masao Nagayama 1994	K Takano 1992
Selection	1.Is the case definition adequate?	a) yes, with independent validation<br>b) yes, eg record linkage or based on self-reports<br>c) no description	*	*	*	*	*	*	*	*	*	*
	2.Representativeness of the cases	a) consecutive or obviously representative series of cases<br>b) potential for selection biases or not stated	*	*	*	*	*	*	*	*	*	*
	3.Selection of Controls	a) community controls<br>b) hospital controls<br>c) no description	-	*	*	*	*	-	-	*	-	-
	4.Definition of Controls	a) no history of disease (endpoint)<br>b) no description of source Comparability	*	*	*	*	*	*	*	*	*	*
Comparability	Comparability of cases and controls on the basis of the design or analysis	a) study controls for __ (Select the most important factor.)	age	age	age	age, sex	age, sex	age	age	age	age	age
		b) study controls for any additional factor (This criteria could be modified to indicate specific control for a second important factor.)	*	*	*	*	*	*	*	*	*	*

Exposure	Ascertainment of exposure	a) secure record (eg surgical records)<br>b) structured interview where blind to case/control status<br>c) interview not blinded to case/control status<br>d) written self-report or medical record only<br>e) no description	*	*	*	*	*	*	*	*	*	*
	Same method of ascertainment for cases and controls	a) yes<br>b) no	*	*	*	*	*	*	*	*	*	*
	Non-Response rate	a) same rate for both groups<br>b) non respondents described<br>c) rate different and no designation	-	-	-	-	-	-	-	-	-	-
total scores (S)			7	8	8	8	8	7	7	8	7	7
