# Supplementary material for: Effect of plasma thrombin-antithrombin complex on ischemic stroke: a systematic review and meta-analysis
Source: Syst Rev. 2023 Feb 14;12:17. doi: 10.1186/s13643-023-02174-9 (PMC9930276; doi:10.1186/s13643-023-02174-9)
Supplement: Supplementary file 3 — Additional file 3: Supplementary Table 3. Meta-regression analysis of each study attribute. [file 13643_2023_2174_MOESM3_ESM.pdf]

Meta-regression analysis of each study attribute

meta_es	Coef	Std. Err	z	*p*	95%CI
year	-0.08	0.06	-1.44	0.15	-0.19-0.03
country	0.14	0.73	0.19	0.848	-1.28-1.56
cases	-0.01	0.01	-0.62	0.53	-0.02-0.12
average age	0.23	0.14	1.57	0.11	-0.06-0.51
test method	-1.86	1.36	-1.37	0.17	-4.51-0.81
