# Supplementary figures and images for: Effect of plasma thrombin-antithrombin complex on ischemic stroke: a systematic review and meta-analysis
Source: Syst Rev. 2023 Feb 14;12:17. doi: 10.1186/s13643-023-02174-9 (PMC9930276; doi:10.1186/s13643-023-02174-9)

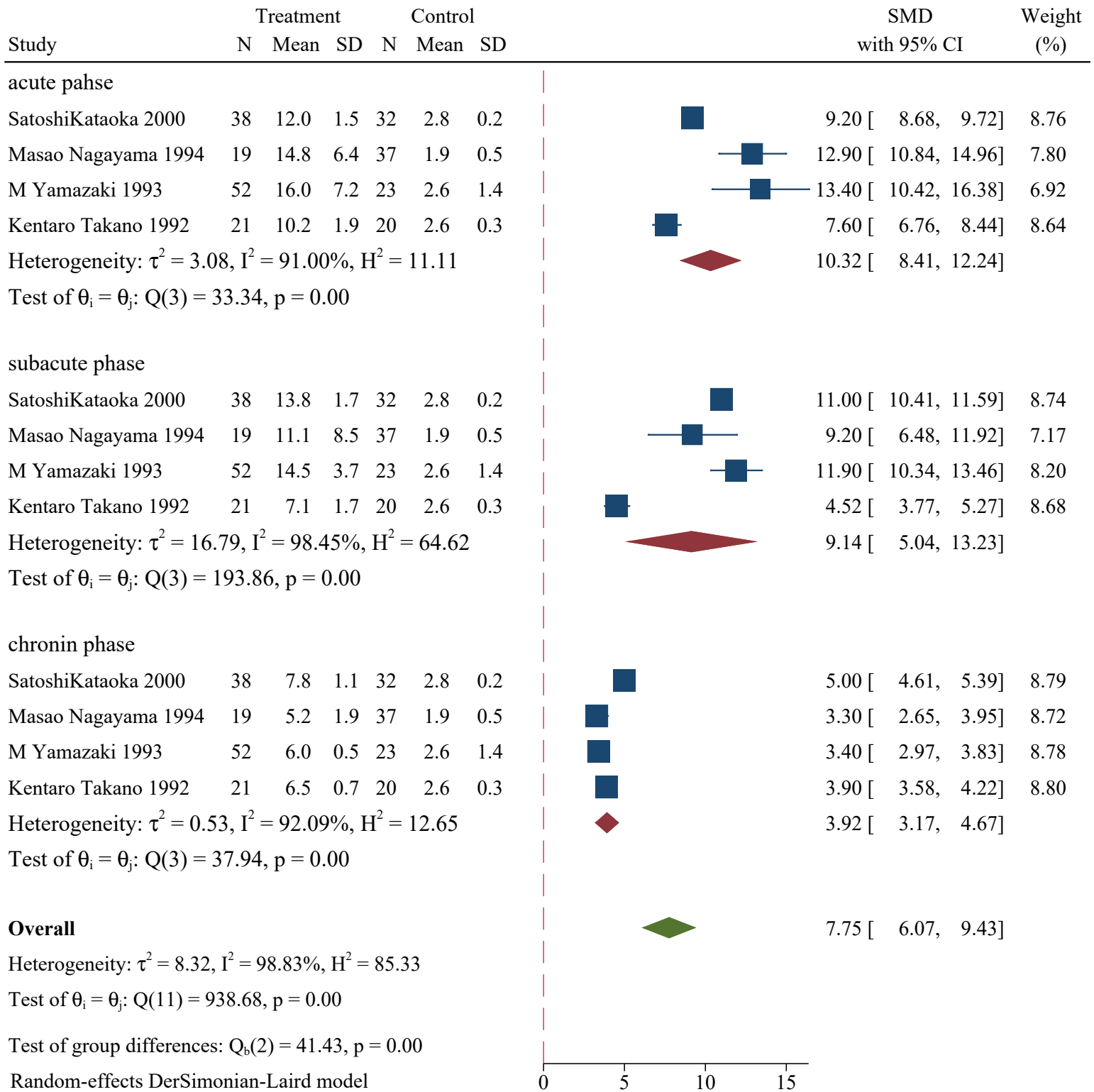

Supplement: Supplementary file 4 — Additional file 4: Supplementary Figure S1. The level of TAT in cardioembolic stroke among the acute, subacute and chronic phases. [file 13643_2023_2174_MOESM4_ESM.pdf]

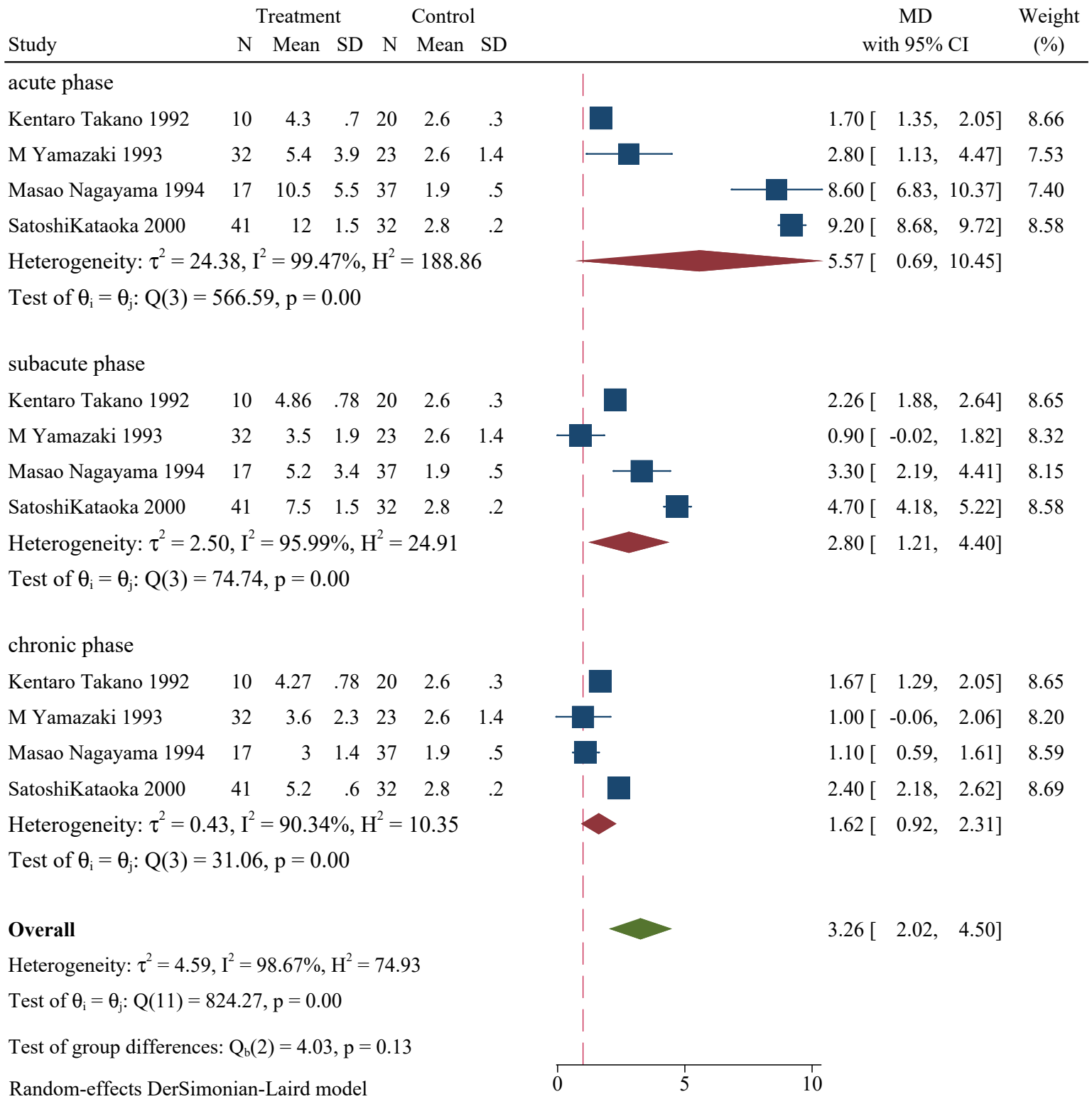

Supplement: Supplementary file 5 — Additional file 5: Supplementary Figure S2. The level of TAT in atherothrombotic stroke among the acute, subacute and chronic phases. [file 13643_2023_2174_MOESM5_ESM.pdf]

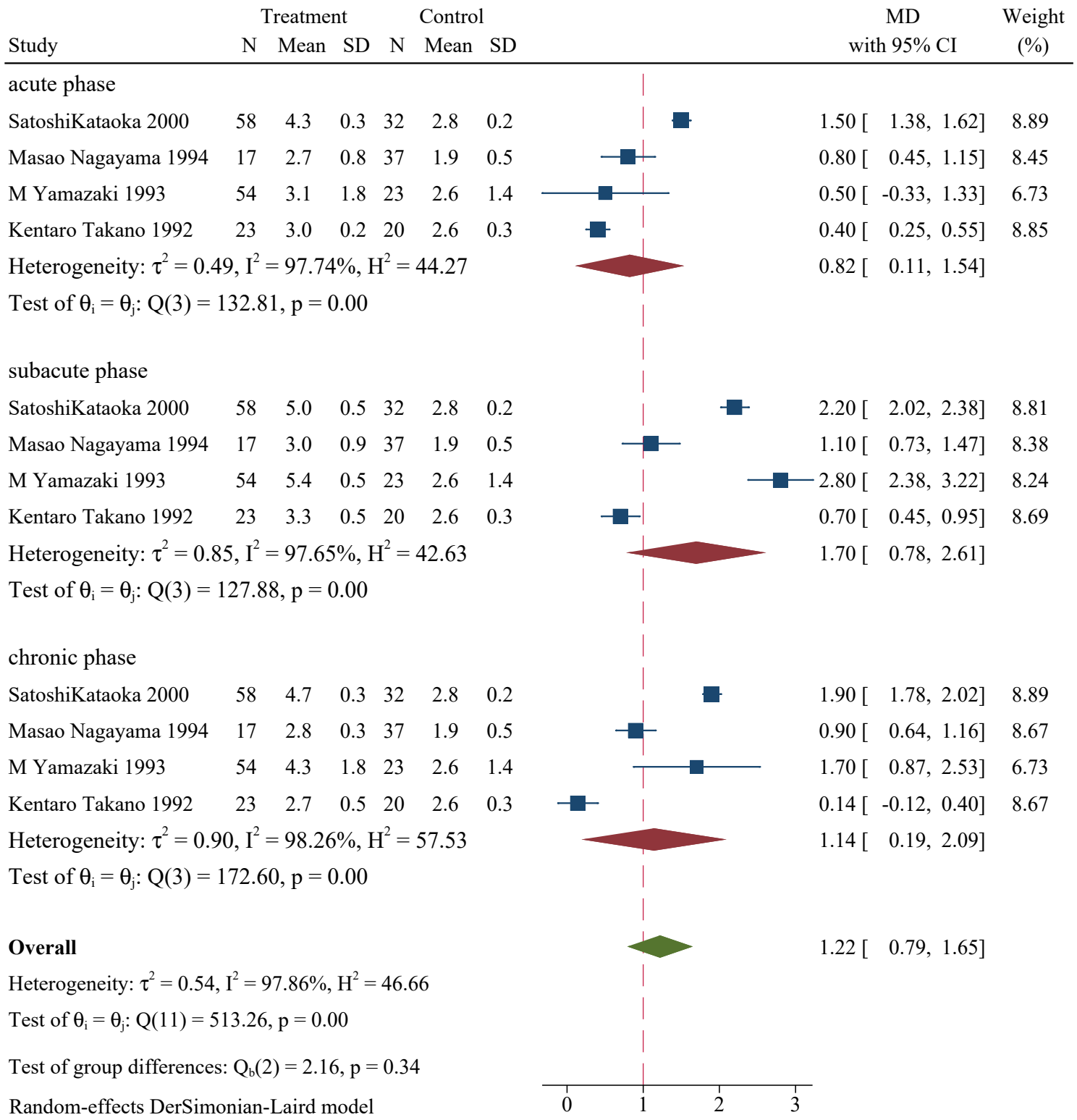

Supplement: Supplementary file 6 — Additional file 6: Supplementary Figure S3. The level of TAT in lacunar stroke among the acute, subacute and chronic phases. [file 13643_2023_2174_MOESM6_ESM.pdf]

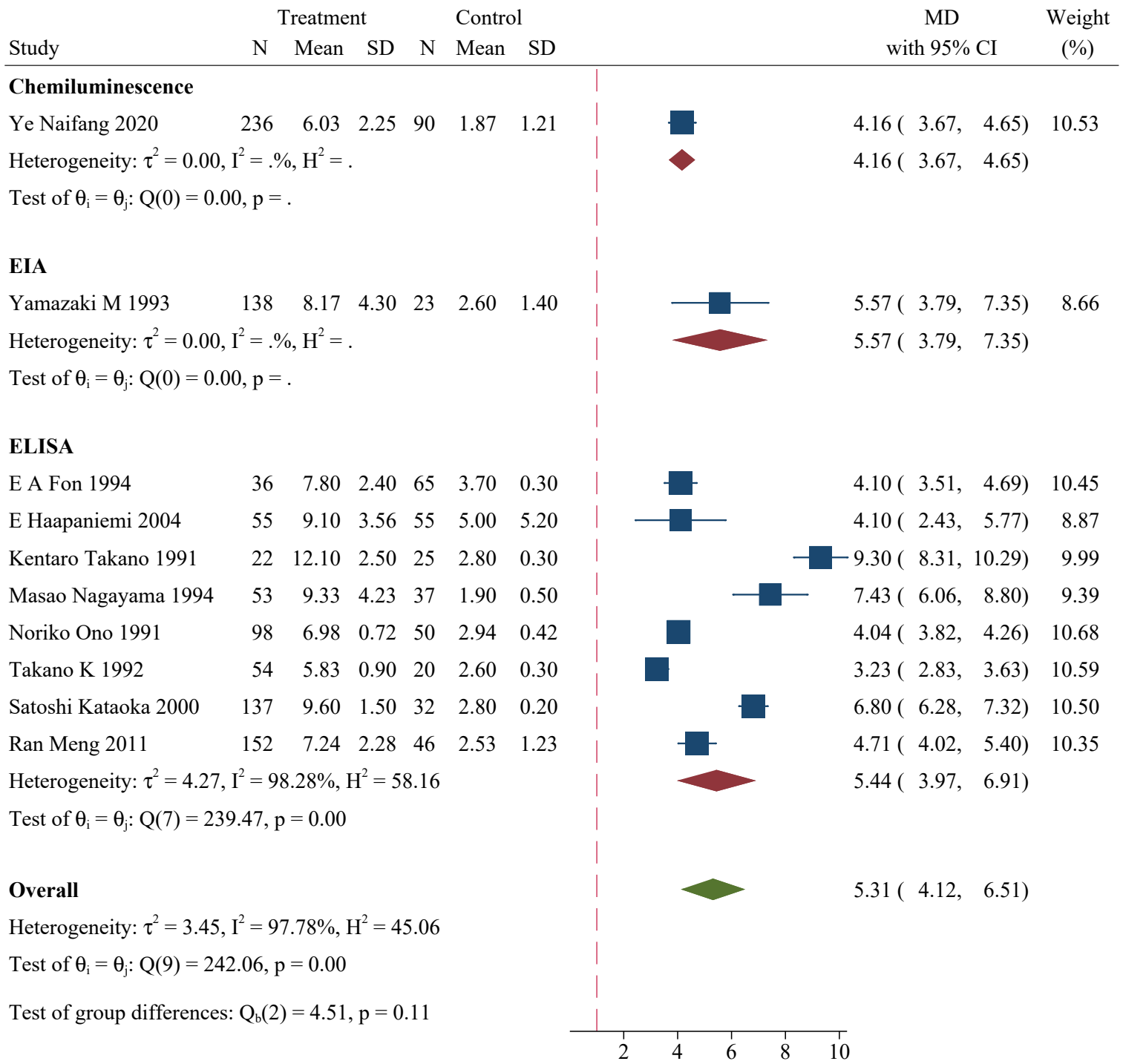

Supplement: Supplementary file 7 — Additional file 7: Supplementary Figure S4. Subgroup analysis of plasma TAT based on the TAT detection method. [file 13643_2023_2174_MOESM7_ESM.pdf]
